# Management of a Patient With an Obstructed Inguinal Hernia With Amyand’s Variety: A Report of a Rare Case With a Review of the Literature

**DOI:** 10.7759/cureus.76828

**Published:** 2025-01-02

**Authors:** B. M. Munasinghe, N. J. A. S. S. Jayasuriya, W. P. N. K. Pathirana, R. V. Paranamanna, M. K. D. H. V. Jayalath, M. W. I. D. Karunarathna

**Affiliations:** 1 Anesthesiology, Base Hospital, Thambuththegama, LKA; 2 Surgery, Base Hospital, Thambuththegama, LKA

**Keywords:** amyand’s hernia, appendix, bowel obstruction, faecolith, lasanoff and basson's classification, mesh repair

## Abstract

Amyand’s hernia is a rare form of inguinal hernia, where the appendix is located within the inguinal sac. A 66-year-old male presented with an obstructed, incarcerated right inguinal hernia. He underwent an emergency herniotomy under spinal anesthesia. Intraoperatively, the hernia sac contained a viable small bowel and part of the appendix. As the appendix appeared mildly inflamed, an appendicectomy was performed, and a synthetic mesh repair was done. He received a postoperative course of antibiotics and was discharged home on day 3 after an uneventful recovery. Histology of the appendix revealed a fecolith in the absence of acute inflammation. He did not have any procedure-related complications on follow-up. Preoperative diagnosis of Amyand’s hernia is a challenge due to nonspecific symptoms and rarity. Lasanoff and Basson classify Amyand’s hernia and provide surgical guidance for its management. Our case belonged to the type 2 category. Management of Amyand’s hernia needs an individually tailored approach rather than strict adherence to conventional guidelines. The presence of an appendicular fecolith in the absence of inflammation provides an interesting area to explore in an Amyand’s hernia.

## Introduction

In 1731, De Garengeot first described the presence of the vermiform appendix in a femoral hernia [1], while the English Surgeon Claudius Amyand documented performing an appendicectomy for an Amyand’s hernia in 1735 [2]. Amyand documented a ruptured appendix in a right inguinal hernia, which led to an enterocutaneous fistula. Like the case he described, it is more common among children [3]. Since then, several cases of Amyand's hernia have been reported in the literature. It is considered a rare clinical entity, with a prevalence of 0.4% to 0.6% [3]. Moreover, diagnosing Amyand’s hernia preoperatively remains a challenge due to its rarity and non-specific symptoms. Most cases are identified intraoperatively [4]. The presence of a complicated appendix in Amyand's hernia is even rarer, with an incidence of around 0.1% [5].

Losanoff and Basson proposed a classification to categorize Amyand's hernia with suggested management for different types [6]. According to their classification, four types of Amyand's hernia are described. Type 1 consists of a normal appendix, and the classification suggests hernia reduction, mesh repair, and appendicectomy in younger patients. Type 2 is described as having an inflamed appendix without abdominal sepsis. Losanoff and Basson have recommended appendicectomy through the hernia. They do not recommend mesh repair for type 2 hernia. Types 3 and 4 contain inflamed appendices with related or unrelated abdominal pathology, requiring laparotomy for type 3 and management as type 1 or 3, depending on the findings in type 4. Several case reports have disputed the guidance suggested by the Losanoff and Basson classification, leading to controversies on ideal management, especially the avoidance of a mesh in type 2 [7, 8].

This case report describes the successful management of a type 2 Amyand's hernia where a synthetic mesh was placed due to the large size of the hernia with risk of recurrence and had an uncomplicated clinical course during the short-term follow-up. It adds to the previous author's argument and highlights the need to revise Losanoff and Basson’s classification based on current evidence. The case is reported in line with Surgical Case Report (SCARE) guidance [9].

## Case presentation

**Figure 1:**
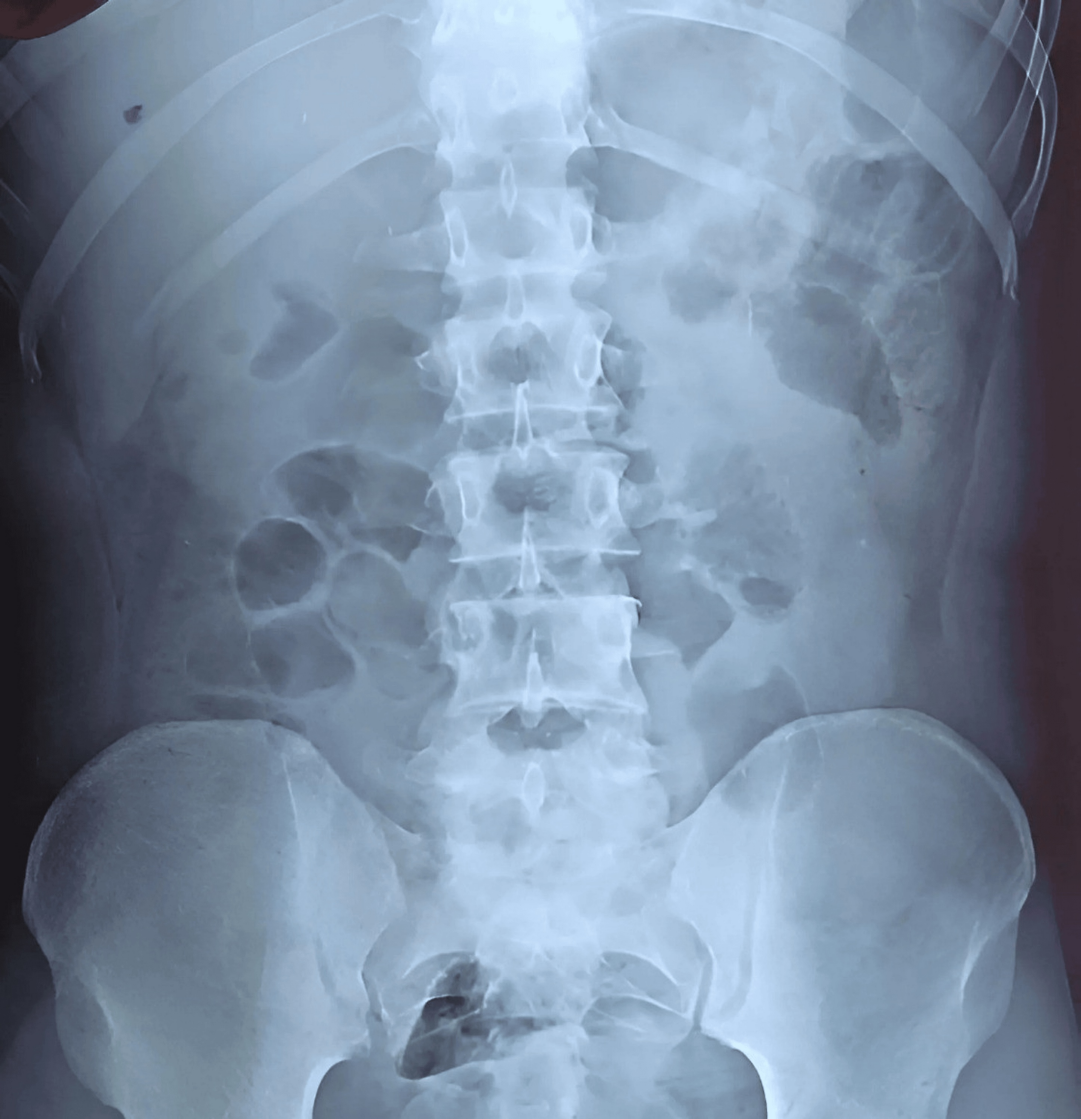
X-ray of the abdomen showing dilated small bowel loops.

A 66-year-old South Asian male, American Society of Anaesthesiology (ASA) stage 3, body mass index 21 kg/m^2^, a farmer by profession, presented to a base hospital in Sri Lanka with a two-hour history of localized right groin pain, non-reducible scrotal swelling and vomiting, with a history of inguinoscrotal hernia for a two-year duration. He had not been regularly followed up for the hernia. The patient was a chronic smoker with chronic obstructive pulmonary disease (COPD) but had no other significant past medical, surgical, or allergy history except for a chronic cough. On examination, he was found to be in severe pain (numerical rating scale 10). His vitals read as follows: blood pressure 182/110 mmHg, pulse 102 beats/minute, respiratory rate 26 breaths/minute, and peripheral oxygen saturation 92% on room air. Bi-basal coarse crepitation was heard during auscultation of the lung fields. On examination of the right groin, an incarcerated inguinal hernia was detected. Out-of-hours ultrasound and CT facilities were unavailable in our hospital and, thus, were not performed. His blood investigations revealed a neutrophil leukocytosis: white cells 12,900 mm^-3^ (reference: 4,000-11,000 mm^-3^), neutrophils 74% (reference: 50%-70%), C-reactive protein 6 mg/L (reference: 3-8 mg/L), and creatinine 0.92 mg/dL (reference: 0.6-1.1 mg/dL) and electrolytes sodium (Na) 136 mmol/L (reference: 135-145 mmol/L) and potassium (K) 5.4 mmol/L (reference: 3.5-5.5 mmol/L). Chest X-ray showed features of COPD with bilateral inflammatory shadows suggestive of infective exacerbation; thus, surgical decompression of the incarcerated hernia was planned under subarachnoid anesthesia. An abdominal X-ray showed a few small dilated bowel loops (Figure 1).

Preoperatively, he was nebulized and received systemic analgesics to relieve his pain.

**Figure 2:**
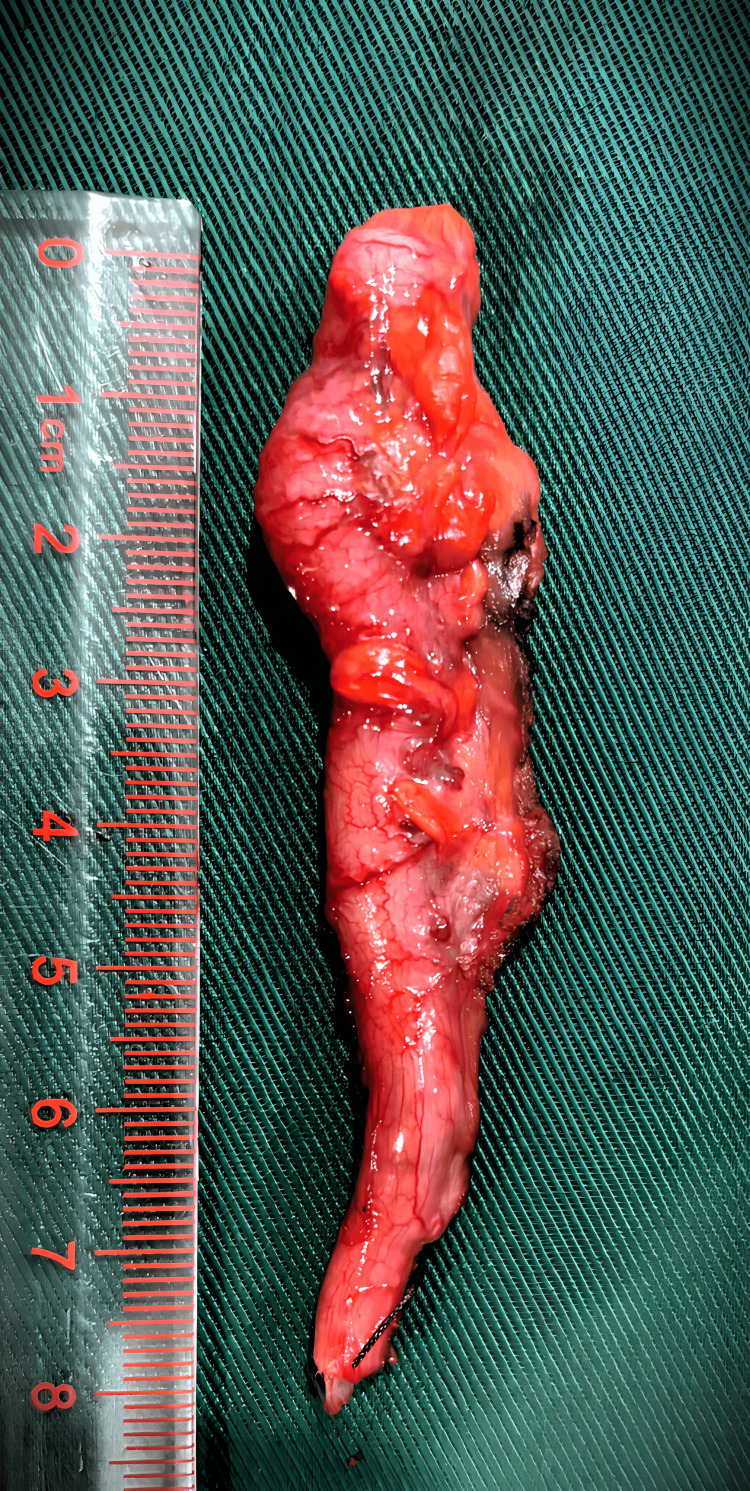
Specimen of appendix resected from Amyand's hernia.

During the surgery, an inguinal incision was made. An indirect inguinal hernial sac was identified. Inside the sac, a loop of small bowel and appendix was found. Due to technical difficulties, an intraoperative image was not obtained. The bowel appeared healthy, and the appendix was mildly inflamed (Figure 2).

**Figure 3:**
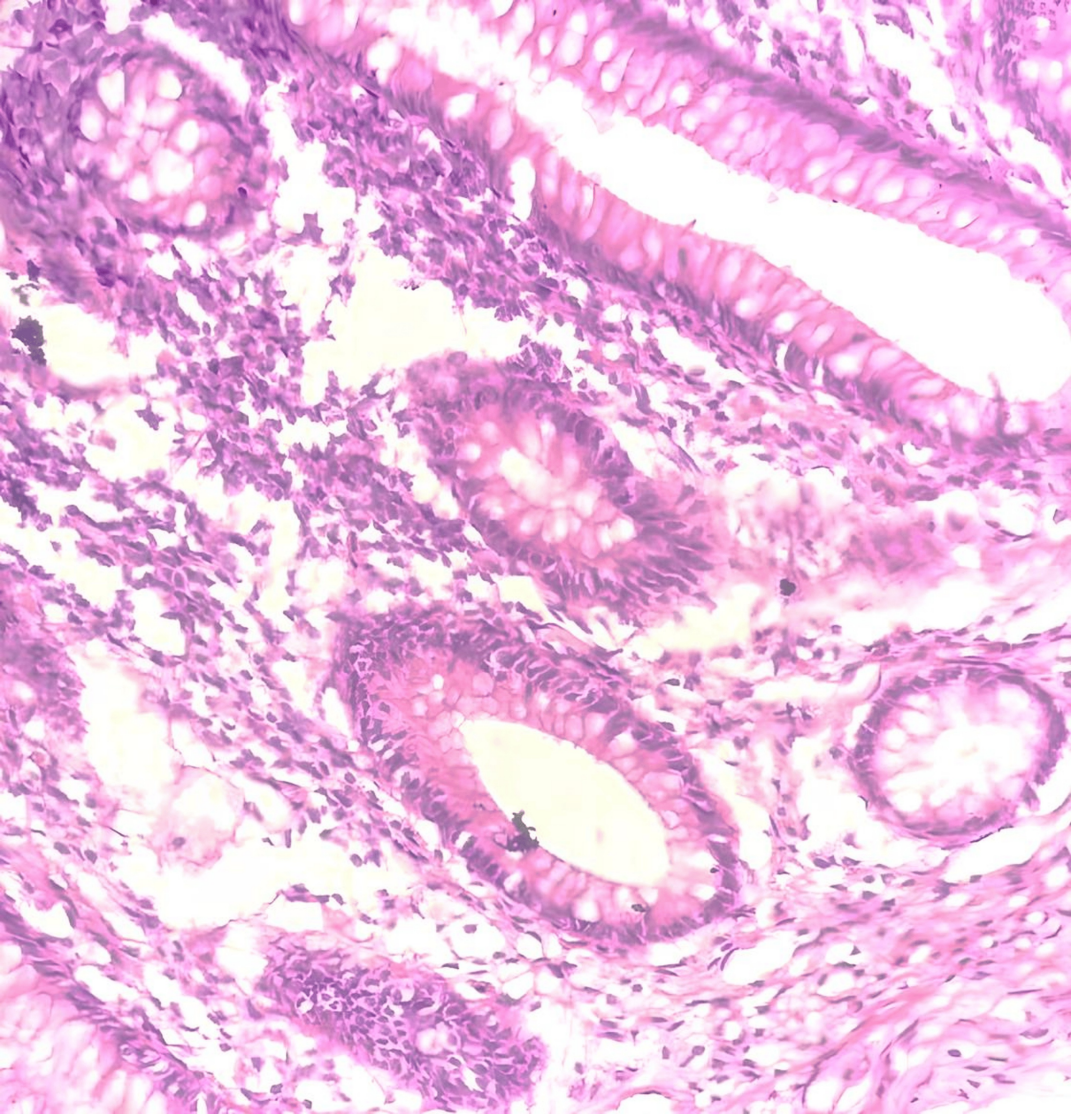
The histology of the appendix showing no significant mural inflammation (H&E, x40). H&E, hematoxylin and eosin

A clinical diagnosis of type 2 Amyand’s hernia was made as the appendix appeared inflamed on macroscopic examination. The cecum was mobilized with careful dissection through the inguinal incision, and an appendicectomy was performed. Hernial sac contents were reduced, and the posterior wall was repaired with 2.0 Vicryl®. Considering the high risk of recurrence and the absence of gross contamination of the surgical field, a polypropylene mesh was placed due to the unavailability of a biological mesh. Routine layered closure was performed. The patient complained of discomfort during the latter stages of closure, so local anesthetic infiltration was done. Postoperatively, the patient received further intravenous antibiotics given the placement of a mesh in the background of a clinically inflamed appendix and was discharged home on day 3. On follow-up at two months, he did not have any surgical complications. The histology of the appendix revealed a luminal fecolith in the absence of significant mural inflammation (Figure 3).

## Discussion

Amyand’s hernia is found between the ages of three weeks to 92 years [10]. The associated mortality and morbidity among adults are 1.8% and 9.2%, respectively [11]. It is thought to consist of one of the following clinical entities: (1) an inguinal hernia with an incidental finding of the vermiform appendix in the hernial sac; (2) an incarcerated inguinal hernia with a strangulated appendix; or (3) an inguinal hernia with an inflamed appendix [7]. Theories explaining the origin of Amyand’s hernia include a lengthy appendix directed toward the groin or loose peritoneal layers and a tortuous, long cecum [12]. The appendix can protrude into the hernial sac and develop complications after the reduced blood supply by compression of the appendix at the external ring [1]. Intraoperative manipulation can similarly initiate inflammation of the appendix [13]. The appendix may be located partially or entirely inside the hernial sac or adhered to the sac wall [13]. It is more common in males and generally occurs on the right side [10]. Left-sided Amyand’s hernia is found in 9.5% of the cases [11]. It is associated with a mobile cecum, gut malrotation, a large appendix, or situs inversus totalis [14,15]. According to the literature, the omentum, cecum, Meckel’s diverticulum, bladder, ovaries, and fallopian tube have been found as contents in reported cases of Amyand’s hernia [16,17].

Hernia-related complications (76%) were the most common reasons to operate on Amyand’s hernia compared to surgery due to acute appendicitis (12%). Appendicular and testicular complications include appendiceal perforation with abscess formation (periappendiceal or intraabdominal), anterior abdominal wall necrotizing fasciitis, epididymoorchitis or testicular abscess, and in situ arterial thrombosis in exceptional cases [18]. The preoperative diagnosis of Amyand’s hernia is difficult. Testicular pathologies (such as torsion, hydrocele, and epididymoorchitis), acute appendicitis, peritonitis, and urosurgical emergencies may mimic Amyand’s hernia [11]. Preoperative diagnosis of Amyand’s hernia only on clinical examination is a challenge due to the rarity and non-specificity of symptoms [4,19,20]. Moreover, surgeons do not usually request preoperative imaging for inguinal hernia unless otherwise indicated [21]. Conversely, as part of the diagnostic workup for acute abdomen, ultrasound and CT may help diagnose Amyand’s hernia preoperatively, although their sensitivity and specificity have not yet been cited in the existing literature [16,20]. A tubular, blind-ended structure with increased vascularity originating from the cecum and extending into the hernial sac is demonstrated on the former. Appendicular congestion, increased wall thickness, and peri-cecal fat density may suggest acute appendicitis. Additional radiological findings with advanced techniques may point toward more complicated pathologies such as gangrenous appendicitis, etc. [22]. Advanced imaging modalities such as CT may be impractical in pediatric patients due to high radiation exposure and are further confounded by the unavailability in low-resource settings [3]. Even at present, the preoperative diagnosis of Amyand’s hernia is around 17.2% [22].

Lasanoff and Basson have classified Amyand’s hernia and its related surgical therapy (Table 1) [6].

**Table 1:** Losanoff and Basson classification.

Type	Description	Surgical management
Type 1	Normal appendix within an inguinal hernia	Hernia reduction, mesh repair, and appendicectomy (young patients)
Type 2	Acute appendicitis within an inguinal hernia, no abdominal sepsis	Appendicectomy through the hernia, primary repair of hernia, no mesh
Type 3	Acute appendicitis within an inguinal hernia, abdominal wall, or peritoneal sepsis	Laparotomy, appendicectomy, primary repair of hernia, no mesh
Type 4	Acute appendicitis within an inguinal hernia, related or unrelated abdominal pathology	Manage as types 1 to 3 hernia, and investigate or treat the second pathology as appropriate

They suggested avoiding using mesh in cases of appendicitis or cavity contamination due to the increased risk of wound and mesh infection and fistula formation from the appendicular stump in types 2 to 4 [16,23]. Conversely, the decision of whether to perform an appendicectomy on a normal-looking appendix in an adult is also controversial, as some argue that it could contaminate an otherwise clean surgical field [13]. The feasibility of mesh repair and the potential use of the appendix as a conduit for urinary diversion are also considered advantages of retaining the appendix when it is not inflamed [24]. Kose et al. reported an incarcerated, non-inflamed appendix in an Amyand’s hernia, requiring appendicectomy due to the presence of fibrous bands between the sac and the appendix [8]. This was a deviation from the classical categorization of Losanoff and Basson. Kuru et al. performed and favored appendicectomy in a type 1 Amyand’s hernia as appendicular complications after minor trauma during the hernial surgery were a concern [25].

As a proponent of appendicectomy for type 1, Quartey et al. described a case of recurrent incarcerated Amyand’s hernia after a normal appendix was kept unresected initially. However, recurrence is extremely rare [24]. Johari et al. proposed appendicectomy for left-sided type 1 hernia due to associated anatomical anomalies and subsequent appendicitis presenting with atypical symptoms [26]. Concerning type 2 hernia, there are case reports of successful mesh repair without immediate and long-term, infection-related complications [4,27,28]. The authors argue that the complications related to the recurrence of large, chronic hernias outweigh the risk of infection and that the risk of infection is less likely, which was the basis for using a mesh in our patient despite the intraoperative clinical finding of a mildly inflamed appendix. The use of biological mesh is proposed in preference to synthetic mesh, further reducing mesh-related infections [16].

There are proposed revisions to the Losanoff and Basson classification. Singal and Gupta reported Amyand’s hernia through incisional hernial defects and classified it as type V (A, B, C) [29]. In their 20-year systematic review of Amyand’s hernia, Mantakis et al. suggested that the appendicectomy may be performed in selected elective hernial surgeries, avoiding contamination of the field, and prosthetic mesh may be used in appendicitis and tissue repairs to be preferred in gross contamination [11]. The presence of a fecolith in an inflamed appendix routinely requires surgical resection to prevent complications such as perforation, abscess formation, and recurrence [30]. However, there is no consensus on surgical management in a non-inflamed appendix when a fecolith is present [31]. It is an interesting area to explore concerning Amyand’s hernia.

The open approach had been the preference for surgery for Amyand’s hernia in the past. Laparoscopy has been performed successfully, offering diagnostic and therapeutic prospects with the advantages of reduced pain scores, early discharge, reduced hospital stays, and better cosmetics [13]. Transabdominal laparoscopy offers better views with a more manageable and safer reduction of the hernial sac into the abdominal cavity while visualizing the entire length of the appendix, maintaining the strength of inguinal canal contents and reducing adhesion formation [32]. It is plausible that laparoscopy may be the choice of surgical access for elective and selected emergency Amyand’s hernial surgeries.

## Conclusions

Management of different types of Amyand’s hernia warrants updated guidance since the classical Losanoff and Basson classification is challenged by evidence. To further strengthen or disprove such quoted evidence, follow-up of postoperative patients who had Amyand’s hernia surgery for an extended period will be beneficial. The patient demographics, type of hernia, institutional experience, and policies will essentially determine the treatment. The presence of a fecolith in a non-inflamed appendix in an Amyand’s hernia is an interesting area to explore.
